# Association of Myopic Optic Disc Deformation with Visual Field Defects in Paired Eyes with Open-Angle Glaucoma: A Cross-Sectional Study

**DOI:** 10.1371/journal.pone.0161961

**Published:** 2016-08-29

**Authors:** Yu Sawada, Masanori Hangai, Makoto Ishikawa, Takeshi Yoshitomi

**Affiliations:** 1 Department of Ophthalmology, Akita University Graduate School of Medicine, Akita, Japan; 2 Department of Ophthalmology, Saitama Medical University, Saitama, Japan; National Eye Institute, UNITED STATES

## Abstract

**Purpose:**

To examine the association of myopia with the visual field (VF) defects in open-angle glaucoma (OAG) using paired eyes to eliminate the effect of unknown confounding factors that are diverse among individuals.

**Methods:**

One hundred eighteen eyes of 59 subjects with myopia (spherical equivalent [SE] ≥ -2 diopter [D] and axial length ≥ 24.0 mm) whose intra-ocular pressure between paired eyes was similar and the mean deviation (MD) of the Humphrey VF test differed by more than 6 dB were included. Refractive errors (SE, axial length) and parameters associated with the papillary and parapapillary myopic deformation (tilt ratio, torsion angle, and β-zone parapapillary atrophy [PPA] area without Bruch’s membrane) were measured in each eye. The paired eyes were divided into worse and better eyes according to the MD of the VF, and parameters were compared between them. Further, multiple linear regression analysis was performed to examine the correlation of the difference in various parameters with the MD difference between paired eyes.

**Results:**

The SE of all eyes was -6.39 ± 2.15 D (mean ± standard deviation) and axial length was 26.42 ± 1.07 mm. MD of the worse and better VF eyes were -13.56 ± 6.65 dB and -4.87 ± 5.32 dB, respectively. Eyes with worse VFs had significantly greater SE, axial length, tilt ratio, and PPA area without Bruch’s membrane than those with better VFs (all *P* < 0.05). In multiple linear regression analysis, the difference of the MD between paired eyes was significantly correlated with the difference in the tilt ratio and PPA area without Bruch’s membrane.

**Conclusion:**

The myopic papillary and parapapillary deformations, but not refractive error itself, were related to the worse VF in paired eyes with OAG. This suggests that myopia influences the severity of the glaucomatous VF defects via structural deformation.

## Introduction

A growing body of evidence supports the idea that myopia is an independent risk factor for the development of glaucoma. Epidemiological studies reported a high prevalence of myopia among glaucoma patients [1, 2]. A recent systematic review and meta-analysis demonstrated an odds ratio of 1.88 for all myopia and 1.77 for low myopia (spherical equivalent [SE] > -3 diopter [D]) in open-angle glaucoma (OAG) [3]. Myopic eyes present characteristic features of the optic disc region including tilt and torsion of the optic disc and parapapillary atrophy (PPA) [1, 4–7]. These morphological changes are considered to be associated with an increased susceptibility to the stress of glaucoma.

Because glaucoma is likely to be a multifactorial disease and the extent that each factor contributes to its development is different in each subject, it is difficult to estimate the contribution of myopia alone. Thus, the influence of myopia on the glaucomatous damage remains unknown. In addition, the myopic deformation of the optic disc and surrounding region varies considerably among individuals even when the refractive errors are similar. Therefore, evaluating myopic deformation and its contribution to the glaucomatous damage among different individuals requires caution.

Comparison of the paired eyes of the same individual has been used in clinical studies because it can eliminate the effects of factors that vary among individuals [8–10]. Within each individual, the paired eyes have the same variables of sex, age, inheritance, and systemic diseases including vascular conditions and diabetes mellitus. They also have the same cerebrospinal fluid pressure. The lamina cribrosas of the paired eyes are reported to have similar configuration, thickness, and material properties [11, 12], which indicate a similar susceptibility to glaucomatous stress. This similarity led us to hypothesize that if the degree of myopia is different in paired eyes of a glaucoma patient with asymmetric visual field (VF) damage, then the VF difference between eyes might be attributed to their myopic difference. The paired-eye study might be an effective method to evaluate the influence of myopia on glaucomatous damage. To our knowledge, there have not been any earlier studies that investigated the difference of myopia in paired eyes with asymmetric glaucoma. The purpose of this study was to determine the association of myopia with the VF defects in OAG using paired eyes to eliminate the effect of unknown confounding factors that are diverse among individuals.

## Materials and Methods

This was a cross-sectional study that was approved by the Review Board of Akita University Graduate School of Medicine. All patients signed an informed consent before participation in the study, and all procedures were in accordance with the tenets of the Declaration of Helsinki.

### Study Subjects

The patients were recruited from the outpatient clinic of Akita University Graduate School of Medicine from June 2011 to July 2015. Each patient underwent a comprehensive ophthalmic assessment, including refraction test, measurement of the best-corrected visual acuity, measurement of central corneal thickness (CCT) and axial length by ultrasound pachymetry (Tomey Corporation, Nagoya, Japan), Goldmann applanation tonometry, slit-lamp biomicroscopy, gonioscopy, dilated fundus stereoscopic examination, color fundus stereo photography (Canon, Tokyo, Japan), spectral domain-optical coherent tomography (SD-OCT) (Spectralis, Heidelberg Engineering GmbH, Heidelberg, Germany), and standard automated perimetry (Humphrey Field Analyzer II 750; 30–2 Swedish interactive threshold algorithm; Carl Zeiss Meditec, Dublin, CA, USA). The intraocular pressure (IOP) was evaluated as the untreated baseline and IOP at the VF examination. The untreated IOP was determined as the average of at least two measurements before the use of IOP lowering medications.

The subject inclusion criteria were as follows: (1) OAG patients with an open iridocorneal angle, glaucomatous optic disc changes such as localized or diffuse rim thinning or retinal nerve fiber defects, and glaucomatous VF defects corresponding to the glaucomatous structural changes. Glaucomatous VF defects were defined as glaucoma hemifield test results outside normal limits, or the presence of at least 3 contiguous non-edge test points within the same hemifield on the pattern deviation plot at < 5%, with at least one of these points at < 1%, which was confirmed on two consecutive reliable tests (fixation loss rate, ≤ 20%; false-positive and false-negative error rates, ≤ 15%). (2) SE was ≥ -2 D and axial length was ≥ 24.0 mm. (3) To minimize the effect of media opacity, the corrected visual acuity was ≥ 20/30. (4) The difference between paired eyes in mean deviation (MD) of the Humphrey VF test was > 6 dB [13, 14]. (5) To ensure that the IOPs of the paired eyes were as close to identical as possible, the difference in the IOPs between paired eyes was ≤ 1 mmHg in untreated baseline and at the VF examination [9]. (6) To ensure that the CCTs of the paired eyes were as close to identical as possible, the difference in the CCT between paired eyes was ≤ 10 μm. Both eyes of each subject had to satisfy the criteria to be included in this paired-eye study.

Subjects were excluded according to the following criteria: (1) Potential subjects with intraocular disease, ocular injury, previous intraocular surgery except uncomplicated cataract extraction and glaucoma surgery. (2) Congenital optic disc abnormalities. (3) Eyes with extremely high myopia due to the difficulty in identifying Bruch’s membrane (BM) opening in SD-OCT images and increased risk of myopic macular changes that might affect the VF.

### Measurement of the Optic Disc Tilt and Torsion

Optic disc tilt and torsion were measured on stereo fundus photographs by two independent observers (Y.S. and T.Y.) who were masked to the patients’ clinical information. Tilt ratio was defined as the ratio between the longest and shortest diameters of the optic disc [6, 15]. Optic disc torsion was defined as the deviation of the long axis of the optic disc and the vertical meridian [15]. The vertical meridian was identified as a vertical line 90 degrees from a horizontal line connecting the fovea and the center of the optic disc. The angle between the long axis of the optic disc and vertical meridian was defined as the torsion angle [15]. A positive torsion value indicated an inferotemporal torsion, and a negative value indicated a superonasal torsion. The absolute values of the torsion angle were used in the analysis to avoid compensation of the positive and negative torsion values.

### Assessment of the PPA Area

The PPA area was assessed in the infrared fundus image shown in the display window of the Spectralis viewer [16, 17]. This enabled us to observe the fundus image and the OCT scan image simultaneously, and pinpoint the location identified in the OCT image on the fundus image. Magnification difference was corrected by adjusting for the curvature of the cornea. Radial scan OCT was performed from the center of the optic disc and included 48 B-scan images, 3.8 degrees apart, in each eye. Each B-scan image was constructed from the 42 averaged frames.

**Fig 1:**
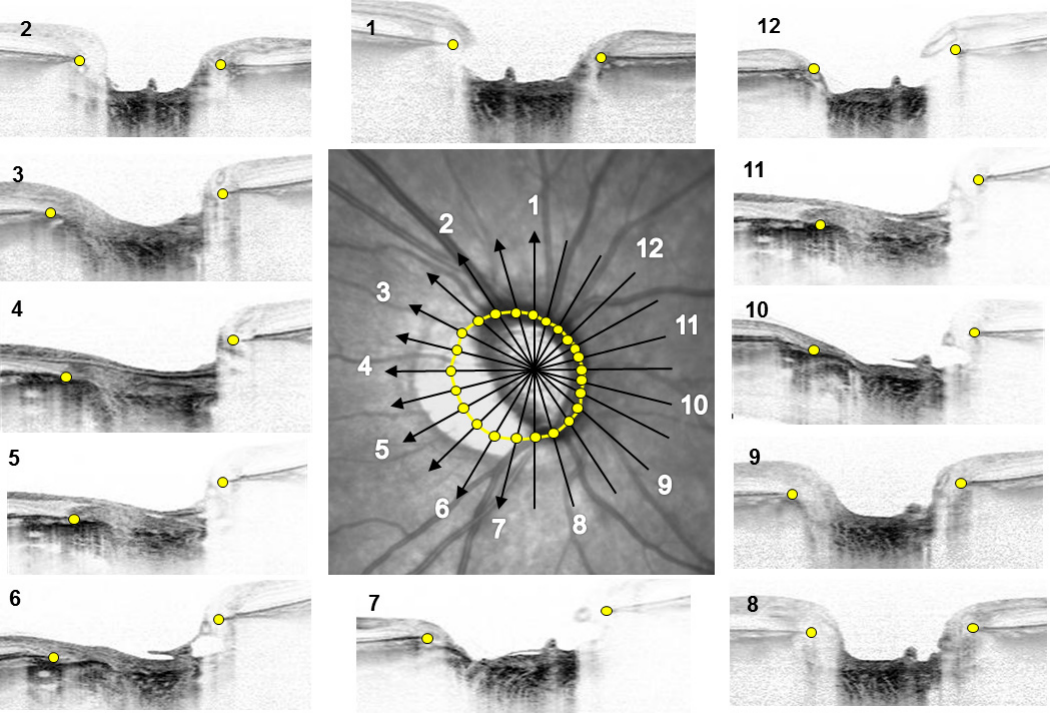
Delineation of Bruch’s membrane (BM) opening area. The central figure is an infrared fundus image of an optic disc with 12 equidistant radial SD-OCT scans. SD-OCT images are presented around the fundus image and placed besides the scan lines that have the identical numbers. The termination of BM was detected in each OCT images (yellow dots), and both sides of the termination were plotted on the scan line. Twenty-four BM terminations plotted on the 12 scan lines were manually delineated, and the extent was defined as the BM opening area (yellow line).

Before assessment, all infrared fundus images were compared with the stereo fundus photographs, and the identification of the β-zone PPA margin and optic disc margin in both images were confirmed. The margin of the β-zone PPA and optic disc were defined as the border between low and high reflectivity in the infrared fundus images. Images were magnified sufficiently to be able to define the borders. The margins were manually delineated using the built-in caliper tool of the OCT system [18]. The β-zone PPA area was defined as the area that subtracted disc area from the area that delineated β-zone PPA margin. The β-zone PPA area was divided into the area with Bruch’s membrane (BM) (PPA_+BM_) and that without BM (PPA_-BM_). The PPA_-BM_ area was obtained as follows. The termination of the BM was identified in 12 equidistant radial SD-OCT images (Fig 1), and both sides of the termination were plotted on the scan line. Twenty-four BM terminations plotted on the 12 scan lines were manually delineated, and the extent was defined as the BM opening area. The PPA_-BM_ area was obtained by subtracting the disc area from the BM opening area. The PPA_+BM_ area was obtained by subtracting the PPA_-BM_ area from the β-zone PPA area. When the image quality of the radial B-scan used for identifying BM termination was suboptimal, a neighboring image was used. When the quality of both neighboring images was suboptimal, the eye was excluded.

### Data Analysis

The paired eyes were divided into worse and better eyes according to the MDs of the Humphrey VF test performed within three months of the OCT examination. The comparison was performed between worse and better VF eyes for the refractive errors (i.e., SE and axial length) and parameters associated with the myopic deformation of the optic disc region (i.e., tilt ratio, torsion angle, and PPA_-BM_ area). Comparisons were performed using paired *t* tests. Multiple linear regression analysis was performed to examine the correlation between paired-eye differences in MDs and various parameters. Inter-observer reproducibility of the measurement of tilt ratio, torsion angle, β-zone PPA area, and PPA_-BM_ area was assessed in 30 randomly selected eyes, and the intraclass correlation coefficients (ICC) with 95% confidence intervals (CI) were calculated. The level of significance was set at *P <* 0.05. The statistical analyses, with two-sided *P*-values, were performed using SPBS ver. 9.66 [19].

## Results

Among 129 subjects enrolled, the followings were excluded (some for multiple reasons): IOP difference between paired eyes more than 1 mmHg (n = 39), VF difference between eyes less than 6 dB (n = 16), CCT difference between eyes more than 10 μm (n = 11), unreliable VF test (n = 14), unclear OCT images (n = 6), congenital optic nerve abnormalities (n = 5), and epiretinal membrane affecting the VF (n = 3). The remaining 59 subjects with 118 eyes were included in the analysis. All of the subjects were Japanese.

The ICCs for the measurement of the tilt ratio and torsion angle were 0.962 (95% CI, 0.933–0.992) and 0.956 (95% CI, 0.936–0.988) respectively. The ICCs for the measurement of the β-zone PPA area and PPA_-BM_ area were 0.970 (95% CI, 0.950–0.982) and 0.946 (95% CI, 0.920–0.968) respectively.

For all eyes, the mean SE was -6.39 ± 2.15 D, and the axial length was 26.42 ± 1.07 mm (Table 1). The mean MD was -9.22 ± 7.38 dB. The measured parameters were compared between paired eyes with worse and better VFs (Table 1). The IOP parameters and CCT were similar between two groups. The MD was -13.56 ± 6.65 dB in the eyes with worse VFs and -4.87 ± 5.32 dB in the eyes with better VFs (*P <* 0.0001). In the eyes with worse VFs, the SE was greater, and the axial length was longer in the eyes with worse VFs than for eyes with better VFs (*P* = 0.0085 and 0.0262, respectively). The tilt ratio was higher in the eyes with worse VFs (*P* < 0.0001), while torsion angle did not differ between the two groups. The PPA_-BM_ area and PPA_+BM_ area were greater in the eyes with worse VFs than in the better eyes (both *P* < 0.0001).

**Table 1:** Subject Demographics and Comparison of Parameters between Eyes with Worse and Better Visual Fields.

		Comparison between Eyes with Worse and Better VF		
Parameters	All Eyes (n = 118)	Worse VF (n = 59)	Better VF (n = 59)	*P* Value
Sex (male/female)	37/22			
Age (yrs)	54.5±13.6			
IOP Untreated (mmHg)	19.3±3.3	19.4±3.3	19.3±3.4	0.4179
VF examination (mmHg)	14.3±2.0	14.4±2.1	14.3±2.0	0.2548
CCT (μm)	522.7±28.4	522.7±28.7	522.8±28.7	0.9408
**Mean deviation (dB)**	-9.22±7.38	**-13.56±6.65**	**-4.87±5.32**	**<0.0001**
**Pattern standard deviation (dB)**	10.00±4.81	**12.52±3.40**	**7.47±4.73**	**<0.0001**
**Spherical equivalent (Diopter)**	-6.39±2.15	**-6.62±2.18**	**-6.12±2.12**	**0.0085**
**Axial length (mm)**	26.42±1.07	**26.50±1.11**	**26.33±1.05**	**0.0262**
**Tilt ratio**	1.33±0.26	**1.38±0.30**	**1.28±0.21**	**<0.0001**
Torsion angle (degree)	10.1±5.3	10.6±5.8	9.5±4.8	0.1156
**PPA area without BM (mm**^**2**^**)**	0.60±0.63	**0.70±0.67**	**0.50±0.58**	**<0.0001**
**PPA area with BM (mm**^**2**^**)**	1.53±0.68	**1.67±0.72**	**1.39±.0.62**	**<0.0001**

Statistical analysis was performed using paired *t* test. Data are shown as mean ± standard deviation.

Statistically significant values are shown in bold.

VF = visual field; IOP = intraocular pressure; CCT = central corneal thickness; PPA = parapapillary atrophy; BM = Burch’s membrane.

In the multiple linear regression analysis, the differences in tilt ratio, PPA_-BM_ area, and PPA_+BM_ area were significantly correlated with the MD differences between paired eyes (*P* < 0.05 for tilt ratio and PPA_-BM_ area, *P* < 0.01 for PPA_+BM_ area), while the same correlation was not found for the axial length (Table 2).

**Table 2:** Multiple Linear Regression Analysis with Difference of MD between Paired Eyes as Dependent Variable.

Parameters	Model 1	Model 2	Model 3	Model 4
IOP untreated	-0.0653	-0.0486	-0.0548	-0.0393
VF examination	-0.0221	-0.0583	-0.0391	-0.0029
CCT	-0.1259	-0.1344	-0.1381	-0.1044
Axial length	0.0487			
**Tilt ratio**		0.2703*		
Torsion angle			0.1431	
**PPA area without BM**				0.2617*
**PPA area with BM**	**0.4066**^†^	**0.3583**^†^	**0.4081**^†^	**0.3766**^†^
Adjusted R	0.3545	0.4460	0.3816	0.4408
*P* value	0.0319	0.0045	0.0193	0.0052

Independent variables are the differences of the parameters between paired eyes.

Values are the standard regression coefficient.

Axial length, tilt ratio, torsion angle, PPA area without BM were included in the separate

models because of the high correlation among them.

Statistically significant values are shown in bold.

* *P* < 0.05

^†^ *P* <0.01

Adjusted multiple correlation coefficients (R) are shown in each model with *P* values.

MD = mean deviation; IOP = intraocular pressure; VF = visual field; CCT = central corneal thickness; PPA = parapapillary atrophy; BM = Bruch’s membrane.

**Fig 2:**
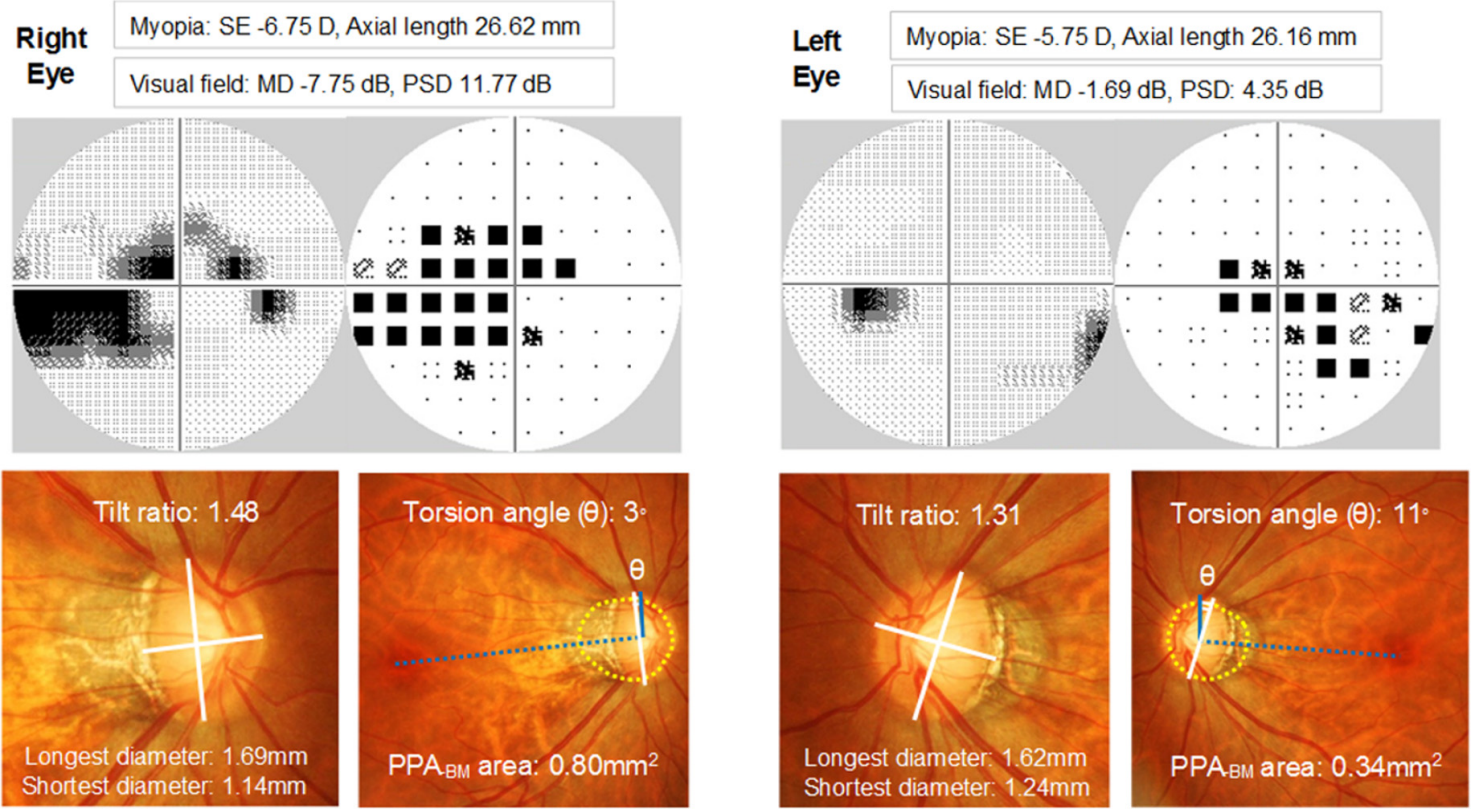
A representative case of myopic open-angle glaucoma patient with asymmetric visual fields (VF). In this 47-year-old female, the right eye with the worse VF had greater myopic refractive errors, tilt ratio, and PPA_-BM_ area than the left eye with the better VF. Humphrey VF data are shown in the gray scale and pattern deviation plot. Solid blue lines are the vertical meridians that are 90 degrees from the line connecting the fovea to the center of the optic disc (dotted blue lines). Yellow dotted lines are the BM openings. SE = spherical equivalent; MD = mean deviation; PSD = pattern standard deviation.

A representative case was shown in Fig 2. In the eyes of a 47-year-old female OAG patient, the myopic refractive errors and myopic deformation of the optic disc region were greater in the right eye with worse VF than in the left eye with better VF.

## Discussion

We evaluated differences in myopic parameters between paired eyes in OAG patients with asymmetric VF defects. The eyes with worse VFs had greater myopic refractive errors and optic disc deformations than those with better VFs based on univariate analysis. Based on multiple linear regression analyses, MD difference between paired eyes was correlated with the differences in tilt ratio, PPA_-BM_ area, and PPA_+BM_ area. Tilt ratio and PPA_-BM_ area are the parameters that present myopic deformation of the optic disc region [7, 20]. The current study, which demonstrated association of tilt ratio and PPA_-BM_ area with the worse VFs, suggested that the susceptibility to the glaucomatous stress was greater in the eyes with severer myopic deformations. The PPA_+BM_ area is speculated to be associated with glaucoma [20]. Our results that showed association between larger PPA_+BM_ area and worse VF defects supports this speculation.

In this study, we used the paired-eye comparisons to estimate the influence of myopia on the glaucomatous damage. This approach minimized the effects of other factors diverse among individuals. Having equal IOP between eyes is particularly important for this purpose because IOP is known to play a role in the development and progression of glaucoma [8, 9, 14]. As used in a previous report, we employed the strictest definition of equal IOP between eyes, in which the IOP difference was ≤ 1 mmHg [9]. This definition was employed for both untreated baseline and IOP at the VF examination to eliminate the effect of IOP as much as possible. CCT is another factor that could affect the development of glaucoma [21]; therefore, we included only paired eyes with similar CCTs.

Myopia is accepted as one of the risk factors for the development of glaucoma [1–3], while its influence on the progression of glaucoma remains controversial [22–24]. A recent meta-analysis summarized studies that investigated prognostic factors of OAG and reported that myopic refractive error was not likely to be associated with the VF progression [24]. We reviewed previous studies that reported the absence of association between myopia and VF progression, and noticed that these studies employed refractive errors (i.e., SE or axial length) as myopic parameters [22, 23]. Although our study investigated the severity of the VF defect, our findings were compatible with these studies in that the refractive error was not associated with the VF status. The differences in refractive errors were not correlated with the differences in the MDs between paired eyes. Instead, the differences in the parameters that presented myopic deformations (e.g., tilt ratio and PPA_-BM_ area) were significantly correlated with the MD difference. This implies that not refractive error itself, but papillary and parapapillary myopic deformations may influence the severity of the VF in OAG.

These findings let us hypothesize two possible processes that caused VF differences between the paired eyes in this study. In the first hypothesis, the eyes with greater myopic deformation developed more VF damage during the childhood progression of myopia, and the VF difference remained after the cessation of the myopic progression. A recent study by Kim et al. demonstrated progressive optic disc tilting with the enlargement of PPA in children who exhibited myopic shift [7]. The structural parameters that were altered during the myopic shift in their study were exactly the same as the ones associated with worse MD in the current study. Doshi et al. reported on a group of young Chinese males who presented non-progressive glaucomatous damage regardless of the use of IOP lowering therapy [25]. The majority of their subjects was myopic and had tilted discs. They speculated that the tilting and associated deformation of the optic discs might develop during the progression of myopia, and these changes could make eyes susceptible to the axonal loss characteristic of the glaucomatous optic neuropathy. Once myopic progression halted and the scleral tension decreased, then the glaucomatous damage would stabilize.

In the second hypothesis, the VF damage progressed after the initial damage created during the development of myopia, and the progression speed was affected by the degree of the myopic deformation of the optic disc region. The parapapillary sclera is considered to be the main stress-bearing tissue of the globe [26]. When optic disc tilt and myopic deformation of the surrounding region develops, it may alter the biomechanics of the parapapillary sclera [27]. With this change, the stretched and flattened temporal part of the optic disc may become the focus of the glaucomatous stress at any level of IOP. In the eyes having a glaucomatous pathology, the optic discs with greater myopic deformation might gain a greater susceptibility to the glaucomatous stress, and it could accelerate the axonal loss and consequent VF damage. It may be more practical to think that the second hypothesis applies to a certain subset of eyes because the VF defect severity and its difference between paired eyes were significant in this study (mean MD of the worse eyes was -13.56 dB and better eyes was -4.87 dB). These differences are not likely to be created only during the development of myopia in childhood. Further longitudinal studies are required to test these hypotheses.

The current study has several limitations. The clinically perceived optic disc margin is not a homogeneous structure because it is composed of some aspect of Bruch’s membrane and the border tissue external to the Bruch’s membrane opening [28]. This might have affected the accuracy of the measurement of the disc area, and subsequent calculation of the PPA_-BM_ area. Also, we employed strict inclusion criteria to assess the influence of myopia on the VF severity, including similar IOPs, CCTs, and asymmetric VFs between paired eyes. This might have excluded a certain portion of potential subjects, and it made the sample size smaller. Further study is needed to examine whether or not the findings are applicable to the general population.

In conclusion, our study showed that of the two eyes of OAG patients, the eyes with greater degrees of myopic deformation presented worse VFs than the eyes with less deformation. The paired-eye design of this study helped rule out the effect of various confounding factors. This finding suggests that myopia has an influence on the VF damage in OAG eyes by way of papillary and parapapillary structural deformation.

## Supporting Information

S1 File Data of paired eyes with better and worse visual fields. Data of the paired eyes with better and worse visual fields of each patient are presented.
(CSV)
